# ADAPTING STAKEHOLDER WALKABILITY/WHEELABILITY AUDIT TOOL IN NEIGHBORHOOD FOR SENSORY AND COGNITIVE DISABILITIES

**DOI:** 10.1093/geroni/igac059.2311

**Published:** 2022-12-20

**Authors:** Kishore Seetharaman, Atiya Mahmood, Bridget Disini, Hailey-Thomas Jenkins, Mike Prescott, Farinaz Rikhtehgaran, W Ben Mortensen

**Affiliations:** Simon Fraser University, Vancouver, British Columbia, Canada; Simon Fraser University, Vancouver, British Columbia, Canada; Simon Fraser University, Vancouver, British Columbia, Canada; Simon Fraser University, Vancouver, British Columbia, Canada; Simon Fraser University, Vancouver, British Columbia, Canada; Simon Fraser University, Vancouver, British Columbia, Canada; University of British Columbia, Vancouver, British Columbia, Canada

## Abstract

Neighbourhood accessibility influences health, social inclusion, and overall wellbeing of older adults. It is important to assess neighbourhood accessibility in relation to the diverse needs and challenges brought on by the intersection of aging and disability, particularly sensory and cognitive disabilities. Given the paucity of neighbourhood audit tools tailored for this population, The user-led Stakeholders’ Walkability/Wheelability Audit in Neighbourhoods (SWAN) tool was originally created for people with mobility disabilities and is now being adapted for seniors with sensory and cognitive disabilities to evaluate functionality, safety, appearance, supportive features, and social aspects in their neighbourhoods. In this paper, we present highlights and key takeaways from the process of adapting the SWAN tool for three user groups: people living with 1) Blindness or low vision, 2) Deafness and hearing loss, and 3) Dementia. Key steps in the iterative tool adaptation process include 1) identifying access needs/challenges for the three user groups based on a literature review, 2) online consultation with stakeholders with lived and/or professional experience (N = 4) to prioritize key access needs/challenges that will be captured through the SWAN tool and review draft versions of the tool, and 3) in-person pilot testing of tools with persons with lived experience (N = 2) in two urban/suburban neighbourhoods in British Columbia, Canada. Reflections of team members and input from stakeholders and pilot participants revealed issues that were addressed in tool development, namely 1) length of audit and participant fatigue, 2) legibility of tool, and 3) tailoring audit to participants’ context and needs.

